# Unravelling the mystery of the ‘minimum important difference’ using practical outcome measures in chronic respiratory disease

**DOI:** 10.1177/1479973118816491

**Published:** 2019-01-03

**Authors:** Linzy Houchen-Wolloff, Rachael A Evans

**Affiliations:** 1 Centre for Exercise and Rehabilitation Science (CERS), NIHR Leicester Biomedical Research Centre – Respiratory, Glenfield Hospital, Leicester, UK; 2 Department of Respiratory Science, University of Leicester, Leicester, UK; 3 Therapy Department, University Hospitals of Leicester NHS Trust, Leicester, UK; 4 Department of Respiratory Medicine, Thoracic Surgery and Allergy, University Hospitals of Leicester NHS Trust, Leicester, UK

**Keywords:** Exercise, outcome assessment, lung disease, MCID, MID, respiratory disease, physical function

## Abstract

It is important for clinicians and researchers to understand the effects of treatments on their patients, both at an individual and group level. In clinical studies, treatment effects are often reported as a change in the outcome measure supported by a measure of variability; for example, the mean change with 95% confidence intervals and a probability (*p*) value to indicate the level of statistical significance. However, a statistically significant change may not indicate a clinically meaningful or important change for clinicians or patients to interpret. The minimum clinically important difference (MCID) or minimally important difference (MID) has therefore been developed to add clinical relevance or patient experience to the reporting of an outcome measure. In this article, we consider the concept of the MID using the example of practical outcome measures in patients with CRD. We describe the various ways in which an MID can be calculated via anchor- and distribution-based methods, looking at practical examples and considering the importance of understanding how an MID was derived when seeking to apply it to a particular situation. The terms MID and MCID are challenging and often used interchangeably. However, we propose all MIDs are described as such, but they could be qualified by a suffix: MIDS (MID – Statistical), MID-C (MID – Clinical outcome), MID-P (MID – Patient determined). However, this type of classification would only work if accepted and adopted. In the meantime, we advise clinicians and researchers to use an MID where possible to aid their interpretation of functional outcome measures and effects of interventions, to add meaning above statistical significance alone.

It is undeniably important for clinicians and clinical researchers to understand the effects of treatments on their patients, both at an individual and group level. In clinical studies, treatment effects are often reported as a change in the outcome measure supported by a measure of variability; for example, the mean change with 95% confidence intervals and a probability (*p*) value to indicate the level of statistical significance for normally distributed data. However, a statistically significant change may not indicate a clinically meaningful or important change for clinicians or patients to interpret. The minimally important difference (MID) has therefore been developed to add clinical relevance or patient experience to the reporting of an outcome measure.

For some outcome measures, such as mortality or frequency of a severe event, the clinical importance is intuitive; very large trials are often needed to demonstrate a statistical difference in these important endpoints, and therefore, surrogate markers (prognostic factors) are frequently developed and investigated . Other treatments may be very important to patients but predominantly impact on health-related quality of life (HRQOL), and a variety of questionnaires^1,2^ have been developed and validated to objectively assess HRQOL in chronic respiratory disease (CRD) over the last 40 years. Clinical interpretation of a meaningful change in a questionnaire score is less intuitive. Similarly, for prognostic factors, it is not always obvious what change is needed to affect mortality or development of severe disease and a ‘number needed to treat’ figure can be calculated. A commonly quoted example to highlight the differences between clinical relevance at a population level versus the individual level is blood pressure reduction whereby an average reduction in 2 mmHg of systolic blood pressure within a population can significantly reduce the frequency of strokes over time,^3^ yet a reduction of 2 mmHg is likely to be trivial to an individual’s risk.^4^

There are consequently different constructs of the minimum important difference including:

1. A statistical difference reflecting a true change has occurred either within a population or in an individual which are usually different values.
2. The difference in a surrogate prognostic factor needed to achieve a reduction in a serious medical event within a population.
3. A meaningful change to patients in measures where interpretation of the change is not intuitive.
4. A change which reflects cost-effectiveness relevant for healthcare systems.
5. A change individuals can detect.

In this article, we consider the concept of the MID using an example of practical outcome measures in patients with CRD.

## Statistical concepts around the ‘minimum important difference’

The minimum important difference (MID) and minimal detectable change (MDC) describe statistical differences without other inference. Distribution-based methods are based on the size of the effect estimate and its relationship to a measure of variability, that is, variance between or within a person’s change.^5^ The most commonly used method is effect size ^6^ represented by the number of standard deviations by which the scores have changed from baseline after the intervention or observation period and is calculated as the mean change divided by the baseline standard deviation (SD). Cohen described a range of effect sizes depending on the comparators. Cohen’s *d* is the most commonly used, where an effect size of 0.2 is considered small, 0.5 moderate, and 0.8 large^6^ for comparing differences between two means of a continuous variable and is most used where the measurement units are arbitrary or where clinical data are insufficient for sample size estimation. Half of the SD of the mean change is another commonly used distribution-based method.^7^ The MDC is defined as ‘the minimal change that falls outside the measurement error’^8^ commonly calculated as MDC = 1.96 × standard error of the measurement (SEM which estimates how repeated measures of a person on the same instrument tend to be distributed around their “true” score) × square root of 2.

**Figure 1.**
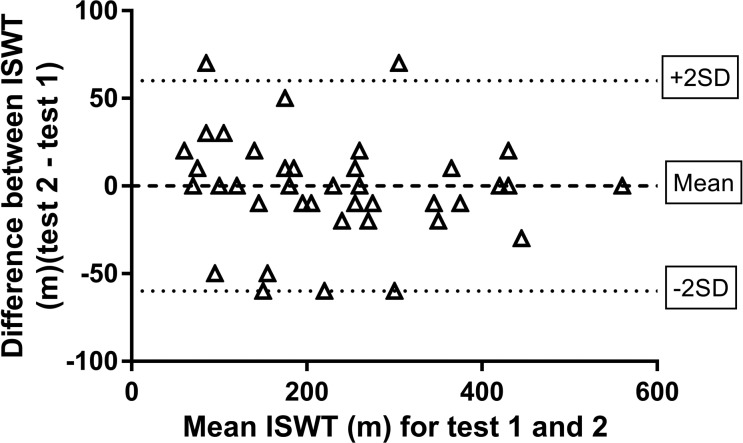
Example data of the repeatability of the incremental shuttle walk test distance. An example from unpublished data where the mean difference for the two tests is zero, so there is no bias. However, there is significant individual variability described by 2 SD approximately 55 metres for the dataset below. These data can aid clinical interpretation within an individual patient; to be 95% confident that a true change had occurred over time or with an intervention an individual would have to improve by >55 metres.

All measurements have an ‘innate’ individual variability even when external conditions are controlled for including disease stability. It is important to understand this concept in daily clinical practice and therefore the magnitude of any natural variation of a test to enable accurate interpretation. These results are generated by multiple repeat testing using the analysis popularized by Bland and Altman.^9^ Although their seminal paper described the difference in measurement between two different methodologies for the same outcome measure, the statistical principles are similar when applied to repeatability. To be 95% confident that a ‘true’ change has occurred, an individual requires to have changed by 2 SD of the mean difference derived by two tests after any learning effect (Figure 1).These concepts are discussed in further detail in an editorial titled ‘Has My Patient Responded?^10^ There may be a bias, that is, the average difference between the two tests is above or below zero, which needs to be taken into account and there may be a relationship between the difference between the two tests depending on the magnitude of the test result known as heteroscedascity.

## The minimum ‘clinically’ important difference

The minimum ‘clinically’ important difference (MCID) aims to add clinical context and describe an important change in a health outcome whether to influence prognosis (living longer) or health-related quality of life (HRQOL) (living better) which are broadly the two important components healthcare is aiming to improve. In the chronic obstructive pulmonary disease (COPD) literature, the MCID gained particular relevance with the development of disease-specific questionnaires to assess HRQOL in the late 1980s. With reference to the Chronic Respiratory Disease Questionnaire (CRDQ), Jaeschke described the MCID as ‘the smallest difference in score in the domain of interest which patients perceive as beneficial and which would mandate, in the absence of troublesome side effects and excessive cost, a change in the patient’s management’.^11^ Subsequently, Schunemann defined the MID as ‘the smallest difference in score in the outcome of interest that informed patients or informed proxies [who can be clinicians] perceive as important, either beneficial or harmful, and which would lead the patient or clinician to consider a change in the management’.^4,12^ The informed proxy may not be able to provide a surrogate rating for outcomes that are intrinsic to the patient, for example, an individual’s perception of dyspnoea. The descriptor ‘clinically’ for the MID was purposefully removed to ensure that the focus was on the patient experience of their daily lives rather than a ‘clinical’ (or clinician’s) view. For the purpose of this review, we use the term ‘MID’ and will describe the particular construct and methodology applied.

## Examples of MIDs using practical outcome measures of exercise performance and function

### Practical outcome measures of exercise performance and function

Exercise performance is often measured in patients with COPD to assess the degree of functional limitation, to prescribe treatment and to measure the outcome of an intervention, for example, pulmonary rehabilitation or pharmacological therapy commonly bronchodilation. It is important for clinicians and researchers to be able to interpret what constitutes a meaningful change in exercise performance to infer whether a treatment has been successful or needs changing and to inform sample size calculations for clinical trials.^12^ Field walking tests are commonly used to assess exercise performance in COPD such as the six-minute walk test (6MWT),^13^ the incremental shuttle walk test (ISWT)^14^ and the endurance shuttle walk test (ESWT).^15^ More recently, practical outcome measures for lower levels of function and frailty have been extensively evaluated in COPD such as the four-metre gait speed (4MGS^16^), five repetition sit to stand test (5STS^17^), timed up and go test (TUG^18^) or a collection of tests as part of the short physical performance battery (SPPB^19^).

### Statistical methodology: Distribution methods

Distribution-based estimates will differ depending on the context, for example, in response to different interventions or in different populations, where the variance is different [more/less heterogeneous].^20^ An example is the calculated MID for the ESWT which was lower for bronchodilation than for pulmonary rehabilitation (PR) due to the differences in effect size: PR results in a much larger magnitude of change than bronchodilation.^21^ An MID is consequently not specific solely to an outcome of interest but also to the context in which it was derived. When calculating sample sizes for research studies or service evaluation, it is therefore important to use an MID relevant to the particular intervention of interest. Table 1 provides examples of MIDs derived by distribution methods for commonly used exercise and functional measures in CRD.

**Table 1.** Described MIDs of commonly used practical outcome measures in CRD with their methods of derivation.

Exercise test	Primary outcome	Population demographics	Suggested MID	Assessment method	Author
6MWT	Distance (m)	Non-CF bronchiectasis, MRC dyspnoea grade ≥1	22.3–24.5 m in response to PR	Anchor (ROC) Distribution (SEM, effect size)	Lee et al.^22^
		COPD, aged 40–75 years, ≥10 pack-year history	30 m reduction in response to no intervention (12-month FU)	Anchor (death and/ or hospitalization)	Polkey et al.^23^
		Severe COPD, bilateral emphysema, suitable for LVRS	18.9–30.6 m in response to pre-LVRS PR	Anchor (SGRQ, SOBQ) Distribution (SEM, Cohen’s and empirical rule effect size)	Puhan et al.^24^
		IPF, baseline 6MWT ≥150 m	24–45 m in response to interferon gamma-1b	Anchor (death and/or hospitalization) Distribution (SEM, effect size)	du Bois et al.^25^
		Stable COPD, eligible for PR	25 m in response to PR	Anchor (patient global rating of change on a 7-point Likert-type scale, ROC) Distribution (effect size, SEM)	Holland et al.^26^
		IPF, diagnosed between 3-36 months prior to enrolment, baseline 6MWT 150-499 m	28 m in response to no intervention (FU for 12-months)	Anchor (SGRQ, FVC %) Distribution (effect size)	Swigris et al.^27^
		Diffuse parenchymal disease (50% had IPF),	29–34 m in response to PR	Anchor (ROC) Distribution (SEM)	Holland et al.^28^
		SOBOE Stable COPD	54 m in response to no intervention	Anchor (social comparison to another patient)	Redelmeier et al.^29^
ISWT	Distance (m)	IPF, eligible for PR	31–46 m in response to PR	Anchor (patient global rating of change on a 5-point Likert-type scale) Distribution (half SD of the change, SEM)	Nolan et al.^30^
		COPD, eligible for PR	35.5 m	Anchor (patient global rating of change for ‘changed’ or unchanged’, ROC)	Evans et al.^31^
		Non-CF bronchiectasis, MRC dyspnoea grade ≥1	35–37 m	Anchor (ROC) Distribution (SEM, effect size)	Lee et al.^22^
		COPD, eligible for PR	47.5 m in response to PR	Anchor (patient global rating of change on a 5-point Likert-type scale)	Singh et al.^32^
ESWT	Duration (sec)	COPD, GOLD stage IV, chronic respiratory failure	186–199 sec in response to PR ± NIPPV (severe hypercapnic COPD)	Anchor (6MWT distance, WR peak, CRQ) Distribution (effect size)	Altenburg et al.^33^
		COPD, mild–severe airflow obstruction	45–85 sec in response to bronchodilation, unable to confidently estimate a value for response to PR	Anchor (patient global rating of change on a 5-point Likert-type scale) Distribution (half SD of the change)	Pepin et al.^21^
Incremental cycle test	WR peak (W)	Severe COPD, bilateral emphysema, suitable for LVRS	4 W in response to pre-LVRS PR (severe COPD)	Anchor (SGRQ, SOBQ) Distribution (SEM, effect size)	Puhan et al.^24^
CWR cycle test	Duration (sec)	Stable COPD, currently not smoking or requiring oxygen	90–101 sec in response to PR	Anchor (patient global rating of change on a 5-point Likert-type scale, CRQ-D, ROC curve) Distribution (effect size)	Puente-Maestu et al.^34^
5STS	Duration (sec)	Stable COPD, eligible for PR	1.7 sec in response to PR	Anchor (patient global rating of change on a 5-point Likert-type scale, ISWT, SGRQ)	Jones et al.^17^
4MGS	Duration (m.s^−1^)	Fibrotic ILD, eligible for PR	0.08–0.11 (m.s^−1^) in response to PR	Distribution (half SD of the change, MDC 95%)	Nolan et al.^35^
		COPD, eligible for PR	0.08–0.11 (m.s^−1^) in response to PR	Anchor (patient global rating of change on a 5-point Likert-type scale, ISWT) Distribution (MDC 95%)	Kon et al.^36^
TUG	Duration (sec)	COPD, eligible for PR	0.9–1.4 sec in response to PR	Anchor (6MWT) Distribution (MDC 95%, half SD of the change)	Mesquita et al.^37^

CRD: chronic respiratory disease; MID: minimal important difference; 6MWT: six-minute walk test; ISWT: incremental shuttle walking test; ESWT: endurance shuttle walk test; CWR: constant work rate; 5STS: five repetition sit-to-stand; 4MGS: four-metre gait speed; TUG: timed up and go; m: metres; sec: seconds; WR: work rate; W: watts; m.s^−1^: metres per second; CF: cystic fibrosis; MRC: Medical Research Council; COPD: chronic obstructive pulmonary disease; LVRS: lung volume reduction surgery; IPF: idiopathic pulmonary fibrosis; PR: pulmonary rehabilitation; SOBOE: shortness of breath on exertion; GOLD: global initiative for obstructive lung disease; ILD: interstitial lung disease; FU: follow-up; NIPPV: non-invasive positive pressure ventilation; ROC: receiver operator curve; SEM: standard error of the measurement; SGRQ: St George’s Respiratory Questionnaire; SOBQ: San Diego shortness of breath questionnaire; FVC%: forced vital capacity percent predicted; SD: standard deviation; CRQ: Chronic Respiratory Questionnaire; CRQ-D: dyspnoea domain of the CRQ; MDC95%: minimal detectable change with 95% confidence.

It has been argued that distribution-based methods should only be employed as ‘temporary substitutes pending availability of empirically established anchor-based MID values’, particularly when there is a lack of consistency in the values derived by various methods.^38^ However, distribution-based methods are useful for understanding whether a likely change has occurred within a population (notwithstanding the lack of clinical interpretation) and also for sample size calculations.

### Anchor-based methods: Patient determined and/or perceived

MIDs developed using anchor-based methodologies are frequently termed MCIDs in the literature. The anchor is typically either another measure usually with an established MID or a patient’s subjective rating of change or ‘global rating of change’ on a 5- or 7-point Likert-type scale: for example, ‘much better’, ‘slightly (or somewhat) better’, ‘about the same’, ‘slightly (or somewhat) worse’, and ‘much worse’^39^. Linear regression may be used to compare the known MID of the anchor with the magnitude of change in the test of interest that corresponds to the established MID. For example, the MID for cycle endurance time was calculated by linear regression of the change in cycle endurance time versus the change in the St George’s Respiratory Questionnaire (SGRQ) and selecting the cycle endurance time which correlated with an improvement of at least 4 units on the SGRQ^40^ (Table 1). This method requires at least a moderate relationship to exist between the anchor and the outcome of interest.^3^

Where the anchor is a global rating of change, the rating may be given by the patient, by the clinician or patients’ proxy, though agreement between what constitutes a meaningful change may differ between these individuals.^12^ Importantly, these anchor-based methods have the advantage of linking the change in a given score to the patient’s perspective. However, patients may place a different value on a particular benefit (inter-individual variation) or even the same patient may place a different value on the same benefit (intra-individual variation) depending on the circumstances.^4^ Many clinical decisions with patients are balanced with risk, for example, the risk of surgery versus the benefits – an individual will have a different perception or interpretation of what the risk is worth versus the potential benefits. The same is true of how ‘important differences’ are assessed. Depending on what a patient has had to ‘invest’ may influence how much they expect to gain for example in the development of the MID for the ESWT the MID was greater for pulmonary rehabilitation (PR) than for bronchodilation.^21^ Participant responses may also depend on their prior experience with the treatments or healthcare outcomes under evaluation.^41^

Another integral part of anchor ratings is a patient may simply be reflecting acuity. They can detect there has been a change, but the change may not be associated with any clinical or patient-reported improvements. It is important that the question for the global ratings of change is precise and easily understood. For example, a question at the end of an intervention could be phrased about how they feel after the intervention or how they performed on an exercise test compared to a previous test. It is likely these questions will yield different results and the latter is more about acuity.

According to a comprehensive review by Copay et al.,^5^ four variations of the anchor-based approach can be described: (1) the within-patients score change, (2) the between-patients score change, (3) the sensitivity- and specificity-based approach, and (4) the social comparison approach. The within-patient score asks patients to rate their improvement in the outcome of interest on a global scale (Likert) described above. The second approach is to compare the difference in response to two adjacent levels on a global rating scale. For example, the MID may be the difference between those who found no change compared to those feeling better. The third approach for calculating MIDs includes sensitivity and specificity analyses. Sensitivity reflects the proportion of patients who report an improvement and whose score exceeds the threshold value, that is, they are ‘a true positive’. Specificity reflects the proportion of patients who report a deterioration and whose score is below the threshold value or a ‘true negative’. A sensitivity of 1 would mean that all true positives were identified, and a specificity of 1 would mean that all true negatives identified. Receiver operating curves (ROCs) can be constructed and the area under the curve (AUC) analysed. The null hypothesis reflects an AUC of 0.5, that is, no better at identifying a true from a false positive than a simple guess. In a systematic review of studies which evaluated the MID of the 6MWT in the elderly or those with chronic respiratory or cardiac disease,^42^ the authors chose an AUC cut-off of 0.7 based on previous expert opinion regarding health status questionnaires.^43^ Where the AUC was at least 0.7 in the latter systematic review, the MID for the 6MWT ranged from 14.0 metres to 30.5 metres. The authors therefore concluded that a change in the 6MWT of 14.0–30.5 metres may be clinically important across multiple patient groups.^42^ Although the previous authors chose an AUC of 0.7, this is arbitrary. Often the cut point is taken from the top left of an ROC curve, but this can vary depending on the specific situation as to how important sensitivity and/or specificity are.

The fourth approach is not widely used where patients are ‘paired up’ to discuss their health then compare themselves to other patients. The MID is the difference between those rating themselves as ‘a little worse’ or ‘a little better’ rather than ‘about the same’ compared to the other patient.^5^ The original MCID for the 6MWT of 54 metres was derived using a social comparison approach^29^ (Table 1); patients were asked to observe others completing exercise and compare their own exercise capacity with that of others.

### A real-life example of the challenges of using MIDs

Another consideration is using the MID of different outcome measures to assess the same intervention for either systematic reviews or service evaluations. The recent UK COPD PR audit data^44^ used the available MID estimates for the 6MWT of 30 m and the ISWT of 48 metres described in a systematic review of field walking tests^45^ to combine and assess the overall results, as both tests are used across the United Kingdom. The 6MWT MID used was derived both by distribution and anchor-based methods; the latter anchor was a global rating of change where any positive change was subsequently combined to analyse two groups: ‘improvers’ or not.^26^ The ISWT MID used was derived by an anchor method using a global rating of change but were described per level of improvement on a 5-point Likert-type scale^32^ A recent description of the ISWT MID using both a distribution and anchor method (similar to that used for the 6MWT) reduced the value to 36.1 metres.^31^ By using the latter MID, the overall results for the audit would have been even better. When rating individual programmes those using the ISWT currently will appear comparatively ‘worse’ than those using the 6MWT.

## MID using clinical outcomes such as healthcare utilization and mortality

The majority of the literature describing the MID of exercise performance tests have used global rating of change scores, other exercise performance tests or HRQOL questionnaires, but few have investigated the association with clinical outcomes such as healthcare utilization or mortality. Investigators of the ‘Evaluation of COPD Longitudinally to Identify Predictive Surrogate End-points’ (ECLIPSE) study reported an annual decrement of 30 metres in 6MWT distance was associated with nearly twice the increased risk of death.^23^ Unfortunately, there was no consistent MID associated with hospitalization.

The MIDs for functional measures in CRD have been carefully described in large cohorts of patients by distribution and anchor methods (Table 1), where the anchors have been commonly used outcomes of PR. Increased 5STS and TUG times have been shown to be significantly correlated with worsening prognosis scores (e.g. BODE/ iBODE indices: Body mass index, airflow Obstruction, Dyspnoea and Exercise capacity)^17,37^ and the 4MGS independently predicts the risk of readmission in older patients hospitalized for acute exacerbation of COPD.^46^ However, further linkage of the MID to clinical outcomes such as falls, fear of falling, fractures, healthcare utilization and mortality would add to the interpretation of these functional measures.

Healthcare utilization is a clinical outcome but also informs health economic evaluation of interventions. ‘Quality-adjusted life years (QALY)’ are often used for cost-effectiveness analyses where a score of 1 = perfect health and 0 = death, anything negative is worse than death; one QALY is equivalent to one year of life in perfect health (a score of 1).^47^ The EuroQol (EQ5D) is frequently used to assess QALYs and has been extended to five levels EQ-5D-5 L. It consists of two parts: a utility index (UI) and a visual analogue scale (VAS) of 1–100, where 0 = ‘the worst health you can imagine’ and 100 = ‘the best health you can imagine’. The UI is calculated from five dimensions: mobility, self-care, usual activities, pain/discomfort and anxiety/depression to generate a five digit number which is then converted to a UI based on EQ5D-5 L value set derived for a particular country.^48^ An MID for the EQ5D-5L has been described for patients with COPD undergoing PR using both distribution and anchor methodologies. The MID for the UI was 0.109 and 0.054, respectively, and the MID for the VAS was 10.1% and 6.99%, respectively.^49^ Similar to other data using anchor methods in the context of PR, very few people rated themselves as worse, so the results are unidirectional for improvement. If using these figures for a sample size calculation, an investigator might be wise to use the larger MID from the distribution method in the secure knowledge that the study would also be powered for a clinically meaningful effect.

## Combining different methodologies of MID

A further method is using a combination of both anchor- and distribution-based methods, assessing the agreement between the values of the MID obtained, and presenting a range of MID values. Puhan et al. used this approach with data from the National Emphysema Treatment Trial (NETT)^24^ to describe MIDs for the 6MWT (18.9–30.6 metres) and the maximal incremental cycle ergometer test (2.2–5.5 watts). A triangulation method to identify a definitive MID which incorporates a distribution-based approach, the latter is patient and professional opinion, and an external (clinically relevant) anchor has been proposed .^50^ Although attractive practically, separate values may be needed depending on the context.

## Other considerations for the interpretation of the MID

There are further caveats with the use of MID of outcome measures. Similar to the distribution method, the anchor method also yields different results across different interventions. For instance, the MID could not be established for the ESWT in the context of PR as the correlations between the anchors and the measured change in ESWT performance were weak.^21^

Endurance tests such as constant power (cycling) or constant speed (walking) tests have their own complications for the assessment of the MID. The relationship between power or speed and duration is curvilinear rather than linear and therefore where the baseline endurance test lies on the curve will affect the responsiveness, independent of any intervention effect^51^. For example, a high power/speed test lasting less than 5 minutes will be less responsive than a test which lasts between 10 and 15 minutes. Although setting endurance tests at a percentage of a maximum test (e.g. 80% of peak oxygen uptake) tries to reduce this effect, where this is positioned on the power-endurance curve is highly variable between individuals.

Perceived global rating of change can be influenced by altered expectations after an intervention. In the original description of the MCID for the ISWT, where patients rated themselves as ‘the same’ after PR was associated with a 20-metre improvement in the ISWT.^32^ It is also important to note that a MID of a change in outcome measure should be bidirectional referring to either an improvement or a deterioration. Due to the effectiveness of PR, it is frequently only possible to be powered to describe important ‘improvements’ due to the low numbers of patients getting ‘worse’ in this context.

It is important to understand the population characteristics for the MID derivation as the MID is likely to be affected by disease severity, type of disease and symptoms (Table 1). Where possible the MID for the particular population of interest should be used. Many of the described MIDs for field walking tests involved secondary care populations and those undergoing PR in patients with moderate-to-severe chronic obstructive pulmonary disease (COPD). The results therefore may not be extrapolated to either mild or early disease, or other CRD, particularly when using anchors regarding how patients feel or whether they can detect a change. For example, patients with severe breathlessness may detect small improvements in ET which may appear trivial to someone with mild disease. For the 6MWT, patients who felt they had improved after PR with a baseline 6MWT <350 metres had a lower change in 6MWT (around 50 metres) compared to those with a baseline 6MWT >350 (around 90 metres).^26^ For the ISWT, there is no relationship between the baseline ISWT and the change in ISWT distance with PR. However, whether the MID was different depending on the baseline ISWT for those that felt they had improved was not assessed.^32^ Whether the absolute change in walk test is described or the relative change is also for debate. For the ISWT, the absolute change was described due to the lack of relationship between baseline walking distance and the change.^32^ The amount of work needed for the same distance will be different across the spectrum of walk distances. For the 6MWT, Holland et al. reported that absolute change was ‘a more sensitive indicator’ than percentage change.^26^

In summary, there are many different approaches to the description and derivation of a MID. It is important to understand how an MID was derived when seeking to apply it to a particular situation. The terms MID and MCID are challenging and often used interchangeably. We propose that all MIDs are described as such, but they could be qualified by a suffix: MID-S (MID – Statistical), MID-P (MID – Patient determined), MID-C (MID – Clinical outcome). However, this type of classification would only work if widely accepted and adopted.

In the meantime, we advise clinicians and researchers to consider the population (severity, symptoms), intervention, and the intended purpose for the MID and seek to match to this as carefully as possible. There are cautions to amalgamation of differently derived MIDs to produce a range, but this is a described method. Whichever methodology is used, the MID is aiding the interpretation of outcome measures and effects of interventions, and should therefore be used where possible to add meaning above statistical significance.
